# Weighted gene co-expression network analysis identifies modules and functionally enriched pathways in the lactation process

**DOI:** 10.1038/s41598-021-81888-z

**Published:** 2021-01-27

**Authors:** Mohammad Farhadian, Seyed Abbas Rafat, Bahman Panahi, Christopher Mayack

**Affiliations:** 1 Department of Animal Science, Faculty of Agriculture, University of Tabriz, Tabriz, Iran; 2 Department of Genomics, Branch for Northwest & West Region, Agricultural Biotechnology Research Institute of Iran (ABRII), Agricultural Research, Education and Extension Organization (AREEO), Tabriz, Iran; 3 Molecular Biology, Genetics, and Bioengineering, Faculty of Engineering and Natural Sciences, Sabancı University, Istanbul, 34956 Turkey

**Keywords:** Agricultural genetics, Animal breeding, Functional genomics, Gene expression, Gene regulation, Genetic association study, Computational models, Data processing, Databases, Gene ontology, Gene regulatory networks, Machine learning

## Abstract

The exponential growth in knowledge has resulted in a better understanding of the lactation process in a wide variety of animals. However, the underlying genetic mechanisms are not yet clearly known. In order to identify the mechanisms involved in the lactation process, various mehods, including meta-analysis, weighted gene co-express network analysis (WGCNA), hub genes identification, gene ontology (GO), and Kyoto Encyclopedia of Genes and Genomes (KEGG) pathway enrichment at before peak (BP), peak (P), and after peak (AP) stages of the lactation processes have been employed. A total of 104, 85, and 26 differentially expressed genes were identified based on PB vs. P, BP vs. AP, and P vs. AP comparisons, respectively. GO and KEGG pathway enrichment analysis revealed that DEGs were significantly enriched in the “ubiquitin-dependent ERAD” and the “chaperone cofactor-dependent protein refolding” in BP vs. P and P vs. P, respectively. WGCNA identified five significant functional modules related to the lactation process. Moreover, *GJA1*, *AP2A2*, and *NPAS3* were defined as hub genes in the identified modules, highlighting the importance of their regulatory impacts on the lactation process. The findings of this study provide new insights into the complex regulatory networks of the lactation process at three distinct stages, while suggesting several candidate genes that may be useful for future animal breeding programs. Furthermore, this study supports the notion that in combination with a meta-analysis, the WGCNA represents an opportunity to achieve a higher resolution analysis that can better predict the most important functional genes that might provide a more robust bio-signature for phenotypic traits, thus providing more suitable biomarker candidates for future studies.

## Introduction

Lactation is a key process for the secretion of milk from the mammary glands. It is a complex and dynamic biological process, which is an essential part of the mammalian reproduction system^1,2^. The milk production rate in most mammalians follows a dynamic curve. After an initial increase in milk yield during early lactation, the lactation rate reaches a peak point. Then, production slowly decreases gradually until the end of the lactation process^3^. Therefore, the lactation process can be divided into three distinct stages; namely, before peak (BP), peak (P), and after peak (AP) phases of lactation. The last step of the process is known as lactation persistency^4^. It has been proposed that an increase in production persistency is an alternative approach which can be used to increase total milk production^3^.

Detailed knowledge of lactation biology at the molecular level is inevitable for the identification of direct causative genes responsible for milk production in livestock breeding programs^5^. The different milk composition at each lactation step can be determined by measuring the transcriptional regulation of the underlying genes^3^. Different metabolic and regulatory pathways that produce fatty acids, amino acids, and carbohydrates are also involved in the lactation process, and they may determine the nutritional quality of the produced milk^3^. For instance, casein and whey protein genes are highly expressed throughout all lactation stages in cattle. It has been reported that during the lactation process, transcriptionally-regulated genes are mostly enriched in terms of receptor activity, catalytic activity, and signal transducer activity^6^. Moreover, the regulatory impacts of *JAK-STAT*, *p38 MAPK*, and the *PI3* kinase pathway on lactation processes have been previously reported^6^.

High-throughput whole-transcriptome sequencing technologies, such as microarray and RNA-Seq, produce an efficient and comprehensive description of the gene expression profiles in a given tissue over time^7–10^. The RNA-seq technology has been applied for studying different mammals, e.g., in Assad and Churra sheep^11^, Ghezel sheep^12^, Holstein cattle^13^, Jersey and Kashmiri cattle^6^, Buffalo^14,15^, humans^16^, Holstein and Jersey cows^17^ and goats^18^. However, analyses typically focuses on differentially expressed gene screening, while the degree of interconnection between the involved genes has not yet been investigated. Because the genes with similar expression patterns may be related in term of function, identifying genes with correlated expression can shed more light on their possible functions^19^. The weighted gene co-expression network can be constructed using the WGCNA algorithm^20,21^. The WGCNA has been used to dissect the feed efficiency of dairy cattle^22^, the milk transcriptome of buffalos^15^, and the liver and muscle transcriptome of lambs^23^, thereby highlighting the power of the co-expression networks to provide deep insights into these complex processes. In our previous study, involving the meta-analysis of milk microarray data from *Rat*, *Wallaby*, and *Bos Taurus*, we identified 31 genes involved in the lactation process^4^. Overall, we found that the candidate genes frequently enhanced cell immunity and growth systems^4^.

In the current study, we first performed a transcripotme meta-analysis to identifiy master-key responsive genes involved at the three stages of the lactation process. Then, the results of the meta-analysis were integrated into system biology approaches, i.e., weighted gene co-expression network analysis, and machine learning models to identify functional modules along with hub genes in each module.

## Results

### Meta-analysis

A number of studies related to the lactation process were selected. Our objective was to identify differentially-expressed genes (DEGs) to explain the transcriptome variation across different lactation stages. Therefore, we performed a meta-analysis of differentially expressed genes. A total of five studies, covering 79 samples, were selected for the meta-analysis. The samples were divided into BP, P, and AP to identify DEGs; each period included 26, 24, and 29 samples, respectively. The range of raw sequence reads per sample was 22.9 to 60.4 million (Supplementary Table S1). Meta-analysis provided information on the number of DEGs, the number of genes that are declared DEG in the meta-analysis that were not identified in any of the individual studies or integration driven discoveries (IDD), and the number of genes that are identified as DEG in individual studies but not in the meta-analysis (loss genes). The results of the meta-analysis performed using two methods are presented in Table 1.

**Table 1:** Results of meta-analysis of RNA-Seq data using Fisher and Invorm methods.

	Common genes	DEG	Fisher method			Invnorm method				DOWN
			DE	IDD	Loss	DE	IDD	Loss	UP	
BP vs. P	14,122	104	103	10	0	73	10	30	78	26
BP vs. AP	13,738	85	83	10	0	57	8	24	21	64
P vs. AP	13,738	26	24	9	0	10	9	11	9	17

DE: corresponds to the number of differentially expressed genes.

IDD: Integration Driven discoveries (the number of genes that are declared DE in the meta-analysis that were not identified in any of the individual studies).

Loss: the number of genes that are identified DE in individual studies but not in meta-analysis.

Table 1 indicates that the *p* value technique combined with Fisher and inverse normal methods for BP vs. P, BP vs. AP, and P vs. AP comparisons give 103 and 73, 83 and 57, 24 and 10 DEGs, respectively. The number of new DEGs (IDD) identified using Fisher and inverse methods were 10:10, 10:8, and 9:9, respectively. A total of 104 DEGs (78 up-regulated and 26 down-regulated), 85 DEGs (21 up-regulated and 64 down-regulated), and 26 DEGs (9 up-regulated and 17 down-regulated) were found in BP vs. P, BP vs. AP, and P vs. AP comparisons. The list of DEGs in BP vs. P, BP vs. AP, and P vs. AP comparisons is presented in Supplementary Files 1–3, respectively.

### Functional analysis of meta-genes

**Figure 1:**
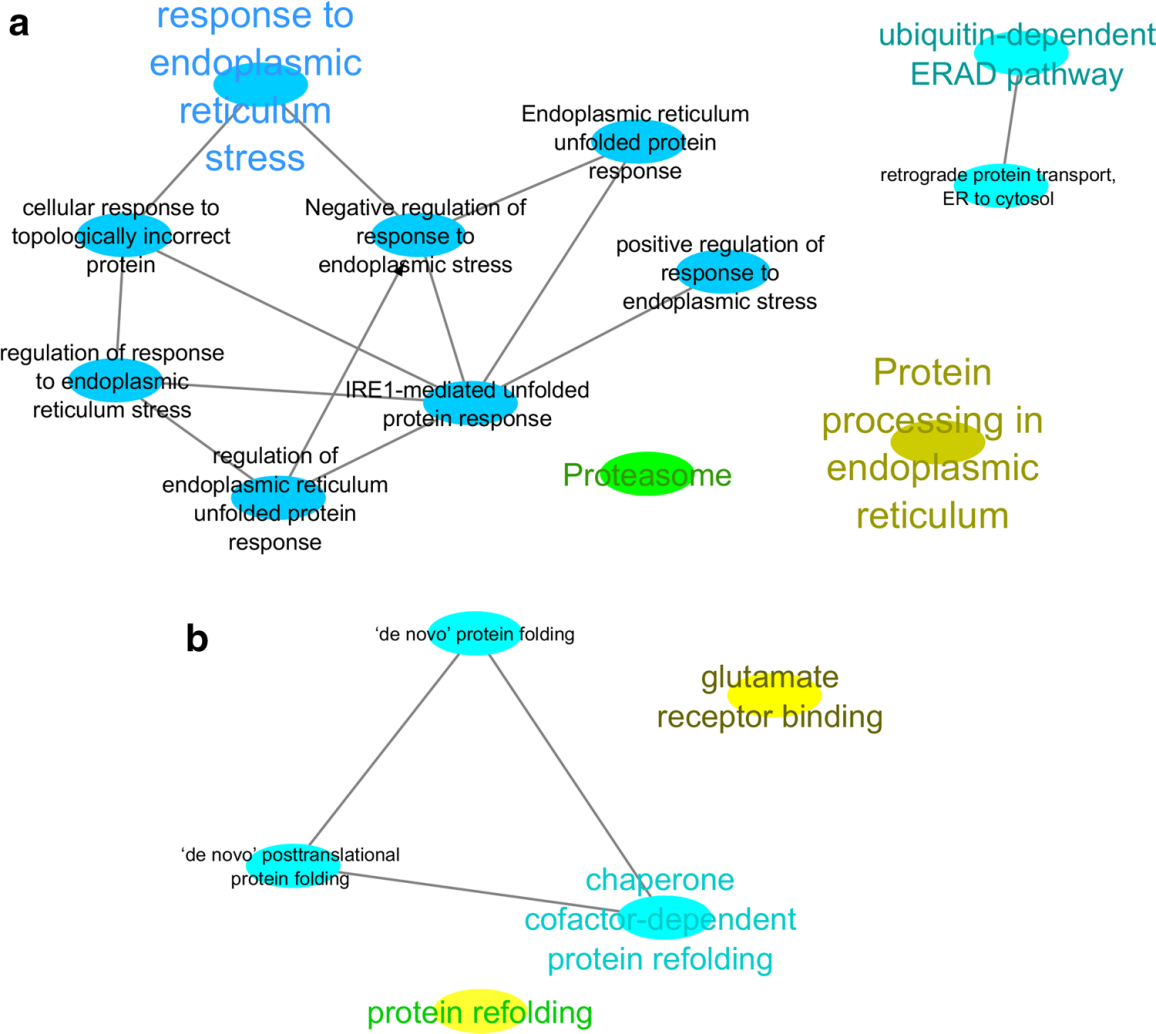
Network visualization of enriched pathways (GO/KEGG)^24^ in the gene signature was performed by ClueGO analysis. (**A**) BP vs. P and (**B**) P vs. AP.

The ubiquitin-dependent ERAD and chaperone cofactor-dependent protein refolding terms were frequently enriched in the BP vs. P and P vs. AP comparisons, respectively (Fig. 1). Enrichment analysis also highlighted ‘protein processing in endoplasmic reticulum’ and ‘response to endoplasmic reticulum stress’ in the BP vs. P comparison. This is while in the P vs. AP meta-analysis, ‘protein refolding’ was the only enriched term for a biological process. Regarding the molecular function category, ‘glutamate receptor binding’ and ‘protein processing in endoplasmic reticulum’ were the enriched terms in the P vs. AP, and BP vs. P meta-analysis, respectively.

The ‘protein processing in endoplasmic reticulum’ is the most important common pathway between associated modules and the DEGs.

### Co-expressed modules related to the lactation process

**Figure 2:**
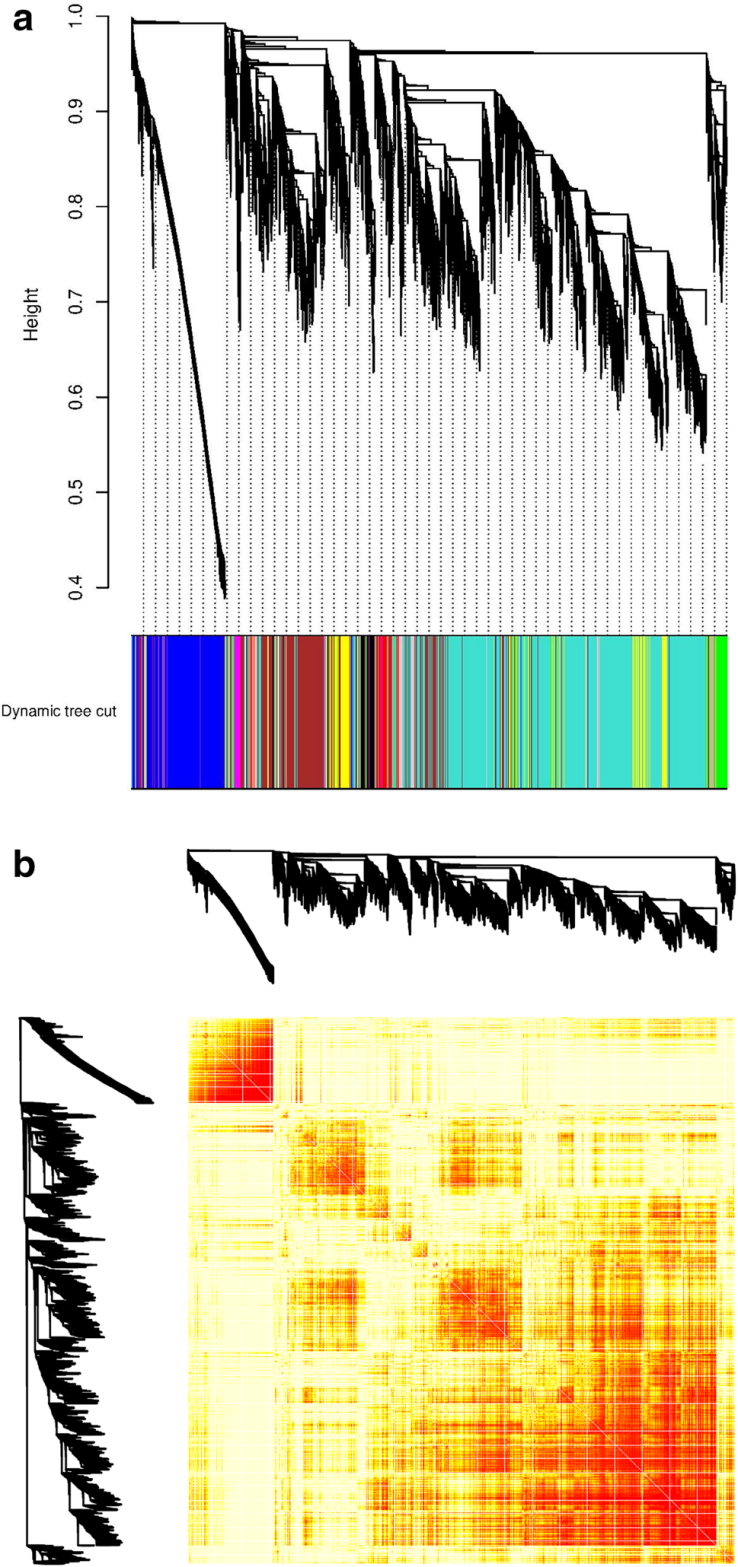
Weighted gene co-expression network analysis (WGCNA) of (**A**) the hierarchical cluster tree of 13,591 meta-genes between the three species. The branches and color bands represent the assigned module; and (**B**) co-expression network modules. In the Topological Overlap Matrix (TOM) plot, the light color represents low overlap and the progressively darker red color represents higher overlap between the genes.

A total of 13,591 meta-genes were identified from the datasets across three species (i.e., *Bos Taurus, Ovis aries,* and *Bubalus bubalis*) (Supplementary File 4). To identify genes that have a strong correlation among the meta-genes, a weighted gene co-expression network analysis (WGCNA) was performed. Using the dynamic tree cutting algorithm, the meta-genes were grouped into 17 modules, which ranged in size from 30 to 5815 genes per modules (Fig. 2A). The hierarchical clustering of the meta-genes, from the three different species across the three different periods of lactation using the topological overlap matrix (TOM), is presented in Fig. 2B.

**Figure 3:**
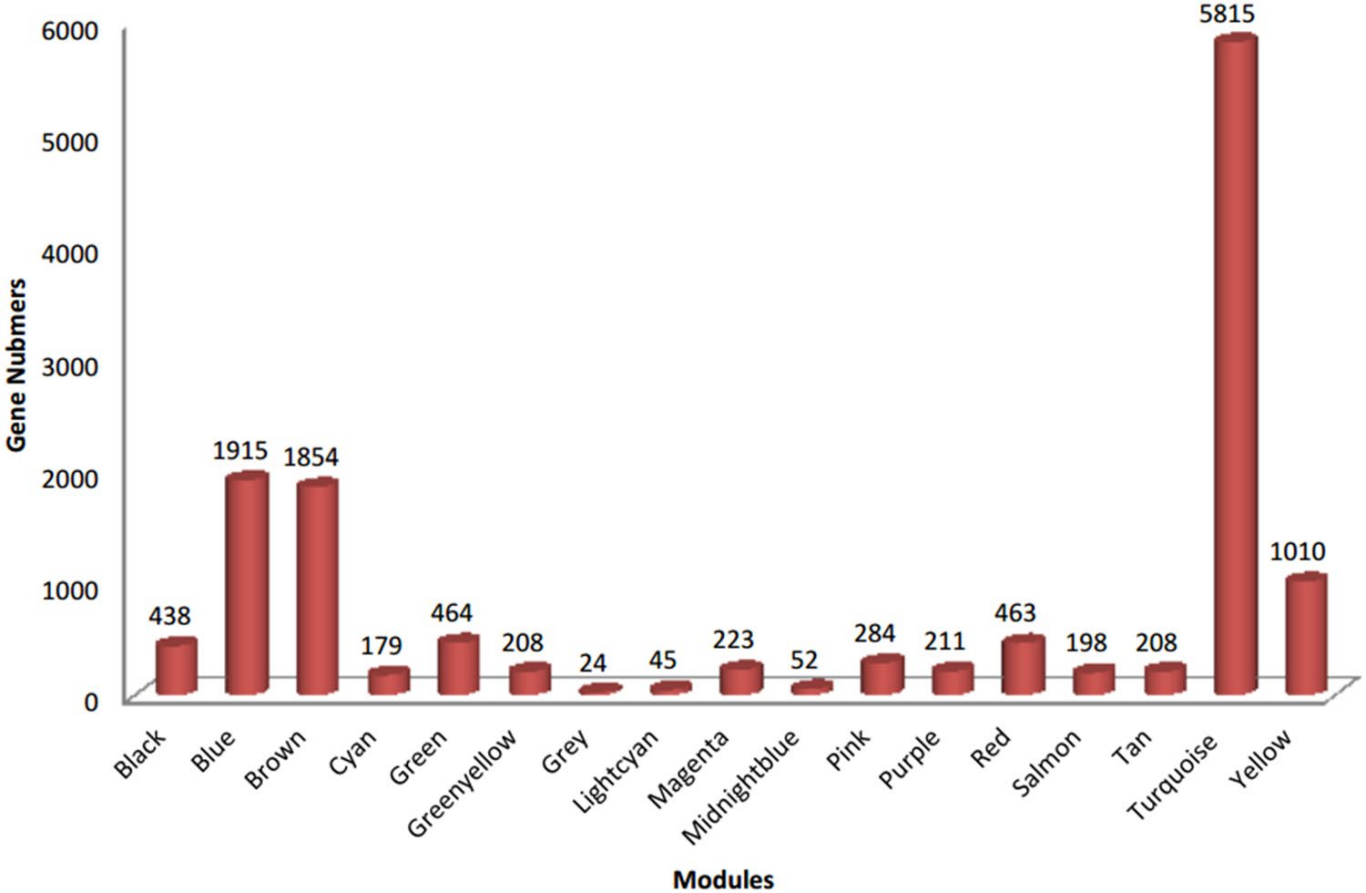
The 17 modules identified by the weighted co-expression analysis (WGCNA) along with the number of genes in each module.

A total of 17 modules were identified (Fig. 3). We show that the three major modules in the co-expresion network include turquoise (n = 5818 genes), blue (n = 1915 genes), and brown (n = 1854 genes).

**Figure 4:**
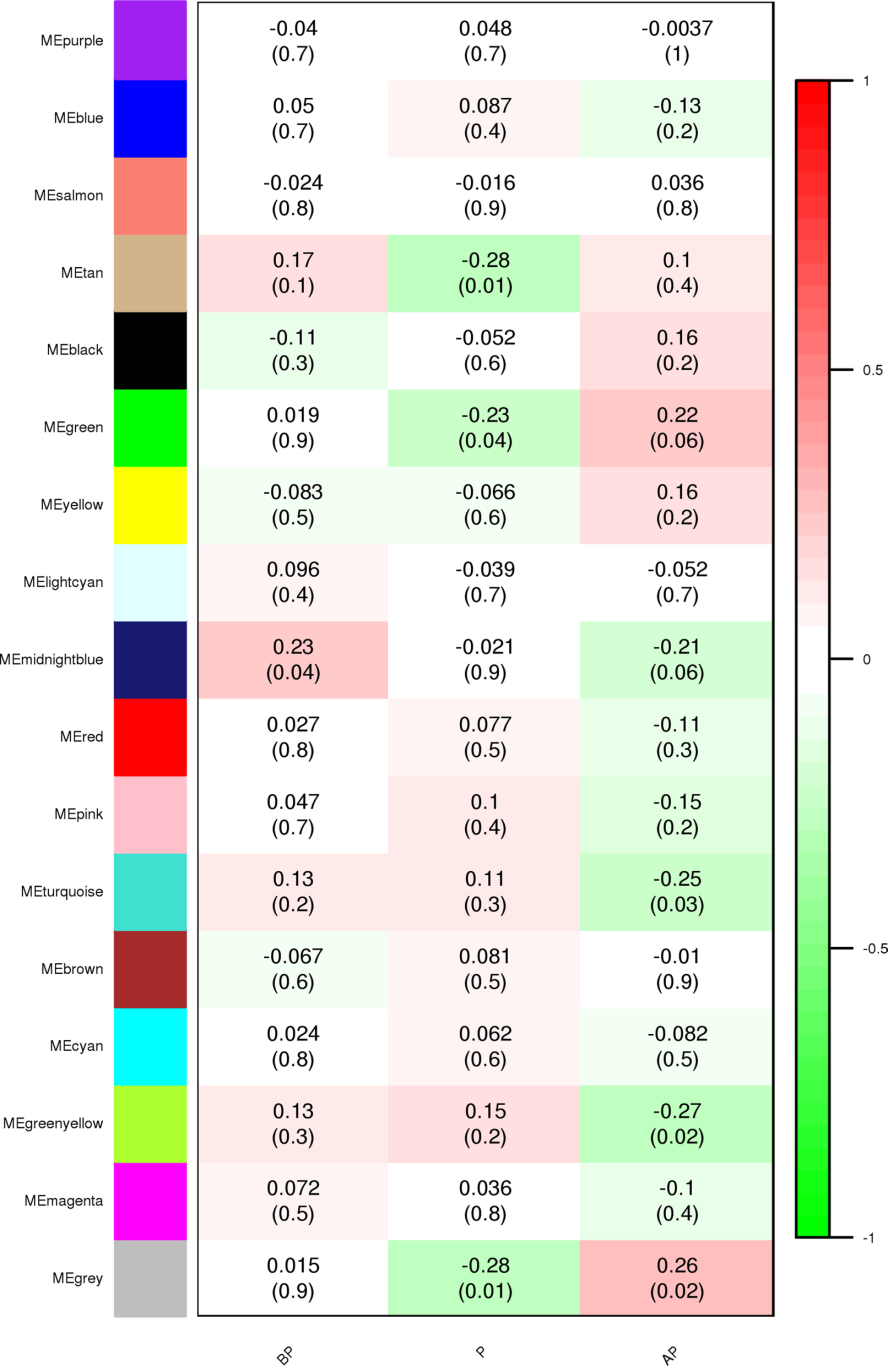
The module trait relationship (*p* value) for identified modules (y-axis) in relation with traits (x-axis). X-axis legend: BP = before peak; P = Peak; AP = after peak.

The 17 functional modules along with their correlation and *p* values are depicted in Fig. 4. It can be observed that the midnight-blue; green, tan; green–yellow, and turquoise modules were specifically significant in BP, P, and AP periods of lactation, respectively. The correlation coefficient and *p* value between the midnight-blue module and the BP period of lactation were 0.26 and 0.04, respectively. The correlation coefficient and *p* value between the green and tan modules and the P period of lactation were − 0.23 (0.04) and − 0.28 (0.01), respectively. Both of these significant modules had a negative correlation with the P period of lactation. The correlation coefficient and *p* value between the green–yellow and turquoise modules in the AP period of lactation were − 0.27 (0.02) and − 0.25 (0.03), respectively.

**Figure 5:**
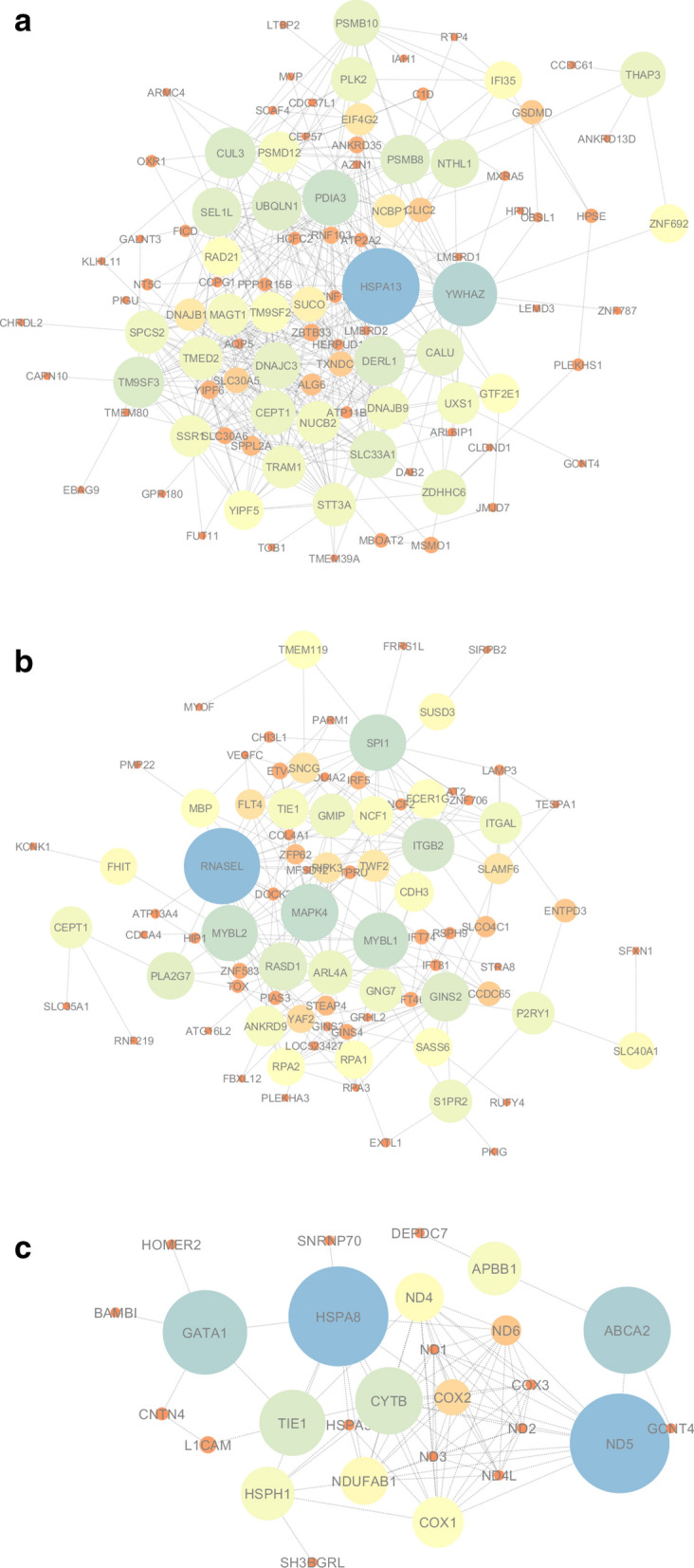
Gene networks for DEGs involved in the lactation process. (**A**) BP vs. P; (**B**) BP vs. AP; and (**C**) P vs. AP comparisons are shown. The mapping strategy of using low parameter values corresponding to bright colors was used for node coloring. The brightest color is green and the darkest color is red. The default middle color is yellow.

The gene network visualization of the gene signatures for the meta-genes in groups BP vs. P, BP vs. AP, and P vs. AP are presented in Fig. 5A–C, respectively. The value of the Betweenness Centrality (BC) is between 0 and 1. The node size in the networks represents the centrality of the corresponding nodes. The significance level for the hub genes is set at BC ≥ 0.1.

Estimated parameters for BP vs. P, BP vs. AP, and P vs. AP networks are presented in Supplementary Files 5, 6, and 7, respectively. Based on the BC values in the BP vs. P comparison, the *HSPA13*, *YWHAZ*, *PDIA3*, *TM9SF3*, and *CUL3* genes were the top five genes. The *RNASEL*, *MAPK4*, *SPI1*, *MYBL2*, and *MYBL1* genes were determined as hubs in the BP vs. AP network. In the P vs. AP network, the *HSPA8*, *ND5*, *ABCA2*, *GATA1*, and *CYTB* genes were the top five hub genes, having the highest value of the BC index.

**Figure 6:**
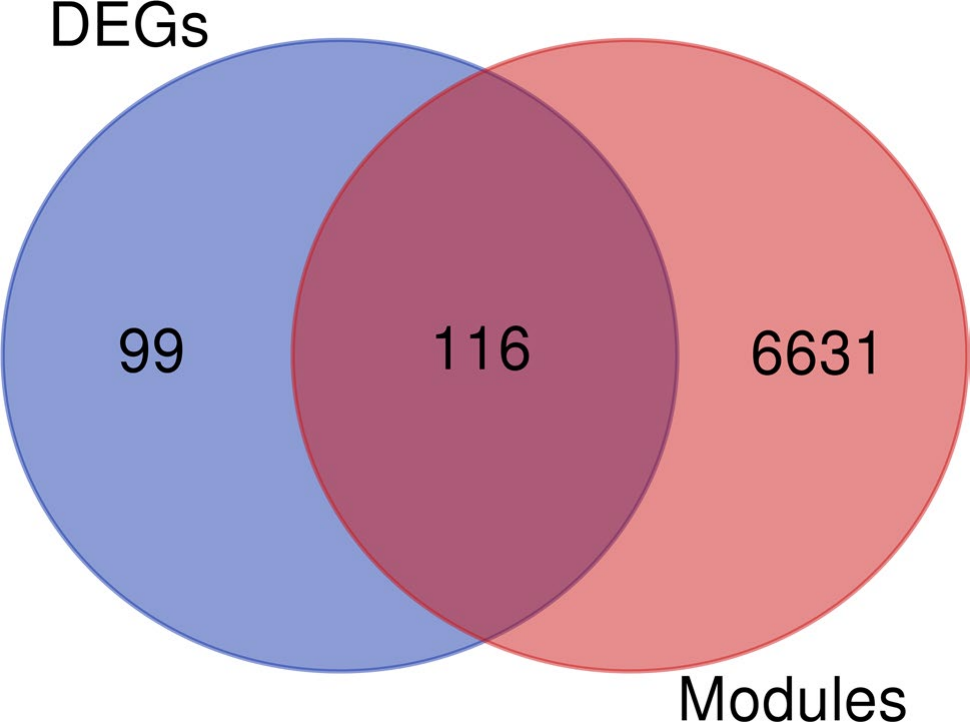
The Venn diagram representing the number of DEGs selected by the meta-analysis and the number of genes selected by the significant modules in the weighted co-expression analysis.

The overlapping DEGs identified through the meta-analysis and the WGCNA (significant modules) approaches are presented in Fig. 6 and Supplementary File 8. Results show that 116 meta-genes involved in significant modules were identified by the WGCNA analysis. Additionally, the hypergeometric test was performed to evaluate the certainty and probability of overlapping between the two approaches, resulting in a value of 0.9839.

### Functional impacts of co-expressed modules

A WGCNA was performed to identify genes that are highly correlated among all meta-genes across different stages of lactation. To perform the functional enrichment of the identified modules, we assigned all the top significant modules in each period of lactation into the ClueGO plugin in the Cytoscape software. The pathway enrichment analysis within the top significant modules indicated that the ‘primary bile acid biosynthesis’, ‘tight junction’, ‘Hippo signaling’, ‘adherens junction’, ‘Rap1 signaling’, ‘phototransduction’, ‘metabolic fatty acid degradation’, and ‘fatty acid metabolism pathways’ were significantly enriched at BP, P, and AP stages, respectively.

### Hub genes identification and validation in co-expressed modules

Five hub genes were extracted for each module (Supplementary File 9). The hub genes identified for each significant module are presented in Table 2.

**Table 2:** Hub genes in significant modules at BP, P and AP stages of lactation.

Lactation stages				
BP	P		AP	
Module	Modules		Modules	
Midnight-blue	Green	Tan	Green–yellow	Turquoise
*FUZ*	*YAP1*	*CAMSAP3*	*P2RX3*	*EIF1AX*
*ZNF32*	*TOM1L1*	*SIX5*	*IQCA1*	*MAGOH*
*ACOT8*	*ESRP1*	*ARHGEF16*	*FAM71F2*	*BAG6*
*WDR18*	*TEAD1*	*TMEM120B*	*CATSPERD*	*POMP*
*KLHDC3*	*SOWAHB*	*GPRC5B*	*LIM2*	*CAPZB*

To validate the identified hub genes, supervised machine learning models were used. Decision Tree (DT) models identified gene bio-signatures that can discriminate different temporal points of lactation. The classification accuracy of the constructed models, based on four criteria, i.e., Information Gain Ratio, Information Gain, Gini Index, and Accuracy, are presented in Table 3.

**Table 3:** Comparison of classification accuracy of constructed Decision Tree (DT) models using different criteria.

Criteria	Accuracy
Information gain	79.03
Information gain ratio	50.63
Gini index	67.85
Accuracy	78.35

**Figure 7:**
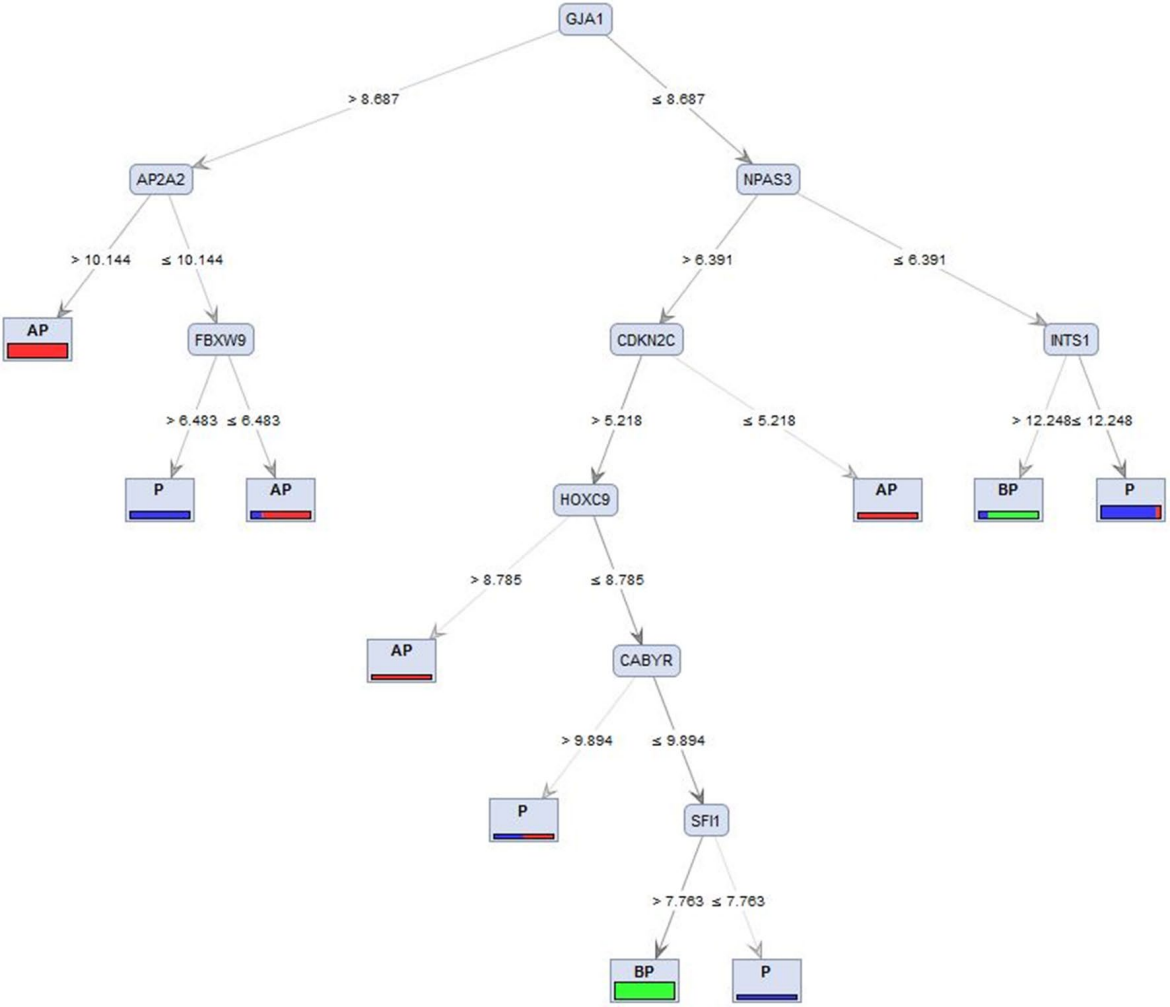
Graphical model of decision tree using Information Gain criterion based on hub genes in three different stages of lactation (Before Peak (BP), Peak (P), and After Peak (AP)).

Results show that DT with the information gain criterion gained the highest (79%) accuracy (Fig. 7). The DT highlighted the role of the top-ranked genes in the classification of different lactation stages based on the expression value of meta-genes^25^. As shown in Fig. 7, the *GJA1* gene has the potential to be considered as a biomarker for the lactation process as it is located at the root of the constructed tree. When the value of the *GJA1* gene was greater than 8.687, and the value of *AP2A2* gene was greater than 10.144, the samples fell into the AP stage. Moreover, if the last feature was equal or lower than 10.144, and the value of the *FBXW9* gene was greater than 6.483, the sample would fall into the P stage. If the last feature was equal or greater than 6.483, samples would fall into the AP stage.

The importance of the *GJA1, AP2A2, FBXW9, NPAS3, INTS1, CDKN2C, HOXC9*, and *SFI1* in turquoise, turquoise, tan, green, turquoise, turquoise, tan, and turquoise modules, respectively, were confirmed using the DT models, highlighting the critical roles of these hubs in the lactation process.

## Discussion

Lactation is known to be associated with a number of physiological and metabolic changes. To gain new insights into the expression and connections of master-key regulatory genes during the lactation process, we analyzed the milk RNA-Seq transcriptome profiling data at different lactation stages using a meta-analysis. Then, we integrated the meta-analysis results into the WGCNA approach. Using the above-mentioned integrative computational and systems biology approach, a set of components responsible for different phases of the lactation process were identified, enabling us to determine which genes play a major role in each period of lactation. Overall, the meta-analysis detected 104, 85, and 26 DEGs for the BP vs. P, BP vs. AP, and P vs. AP comparisons, 73.5%, 25.5%, and 34.6% of which were categorized as up-regulated, while 26.4%, 74.4%, and 65.3% were categorized as down-regulated in BP vs. P, BP vs. AP, and P vs. AP comparisons, respectively.

The ‘response to endoplasmic reticulum stress’ GO term was enriched in BP vs. P periods within the meta-analysis. Milk fat depression studies in laboratory animals^26^ suggest that endoplasmic reticulum (ER) stress plays a role in the regulation of lipogenic pathways in mammary epithelial cells in mice^27,28^. Meta-genes of the P vs. AP comparison indicated that there was an enrichment in the protein refolding biological process term, and that casein is a well-known major component of milk protein^29^. The role of chaperone-like activity^30^ and the aggregation inhibitor function^31^ for casein have been proved to affect other types of milk protein, including b-lactoglobulin^32,33^, a-lactalbumin^32^, and milk whey proteins^34^. Therefore, casein is important for the stabilization of milk whey protein^30^. One of the main functions of the glutamate receptor in rats involves the regulation of the growth hormone^35^. The endoplasmic reticulum synthesizes almost all lipids, including phospholipids and cholesterol. The endoplasmic reticulum (ER) is a critical site for protein, lipid, and glucose metabolism, lipoprotein secretion, and calcium homeostasis^36^. Previous research has demonstrated that intracellular triglyceride droplets, known as cytoplasmic lipid droplets, are secreted into milk as plasma membrane bilayer-coated structures (i.e., milk fat globules)^37^.

Co-expression network analysis of meta-genes identified 17 co-expressed modules across all three stages of lactation. The significant modules identified included 1 (midnight-blue), 2 (green, tan), and 2 (green–yellow and turquoise) modules for BP, P, and AP periods of lactation, respectively.

Primary bile acid biosynthesis is the only enriched pathway found in the midnight-blue module during the BP period of lactation. Bile acids are steroid carboxylic acids derived from cholesterol in vertebrates^38^. It has been demonstrated that cholesterol esters, glycerides, and phospholipids of milk are all made from fatty acids within the mammary gland^38^. Bile acids play an important role in animal husbandry because they promote the digestion and absorption of fat and fat-soluble substances, saving the energy of the animal, promoting animal growth, and thereby improving carcass quality of livestock^39^.

Two significant modules in the P period of lactation are associated with the activation of four pathways, i.e., the tight junction, Hippo signaling, Adherens junction, and the Rap1 signaling pathway. In the mammary gland, during lactation, the tight junctions of the alveolar epithelial cells are impermeable, and, consequently, they allow milk to be stored between nursing periods without leakage of milk components from the lumen. Nonetheless, mammary epithelial tight junctions are dynamic, and a number of stimuli can regulate them^40^. Systemic factors such as progesterone, prolactin, and glucocorticoids along with local factors, such as TGF-beta intra-mammary and pressure play a crucial role in the regulation of mammary tight junctions. On the other hand, the tight junction activation has a negative correlation with milk secretion^40^. The next enriched pathway in the P period of lactation is the Hippo signaling pathway. This pathway plays an important role in the control of organ size of animals, and it operates through the regulation of cell apoptosis and proliferation^41^. Moreover, it has been reported that this pathway has a direct impact on the mammary gland development and the lactation process^42^. Furthermore, cell proliferation and cell differentiation can sustain the growth of the mammary gland and contribute to milk production^4,43^. The adherence junction is another activated pathway found in the P period of lactation. Previous research has confirmed that the proteins of this pathway are involved in breast cancer^44^. This pathway includes a number of intracellular components, such as p120-catenin, β-catenin, and α-catenin^45^. Previous studies have demonstrated that the adherence junction in the epithelial cells aid in their survival during lactation^46^. The last significant pathway in the P period is the Rap1 signaling pathway. Previous research has found that the Rap1 pathway is a pivotal element in mammary epithelial cells^47^. All these enriched pathways in the P period are involved in cell differentiation and proliferation of the mammary gland, and, consequently, they influence milk production.

Two significant modules enriched four pathways (i.e., phototransduction, metabolic pathway, fatty acid degradation, and fatty acid metabolism) in the AP period of lactation. Phototransduction is the conversion of light into a change in the electrical potential across the cell membrane. This process activates some signals, leading to the opening or closing of ion channels in the cell membrane^48^. On the other hand, milk contains many mineral ions, which are regulated by the ion channel control^49^. Three remain as activated pathways, including the metabolic pathways, fatty acid degradation, and fatty acid metabolism, all of which contribute to fat metabolism. In general, metabolic pathways are associated with a series of chemical reactions, such as fat metabolism. In most cases of metabolic pathways, the product of one enzyme acts as the substrate for the next^50^. These enzymes often require dietary minerals, vitamins, and other cofactors to function. In addition, milk components such as proteins (whey 20% and casein 80%), carbohydrates, coated lipid droplets, water, and ions are synthesized and secreted by the mammary gland^51^. Milk fat is considered as one of the most important factors in the quality of dairy product in the dairy industry^51,52^. The main lipid-associated metabolic pathways include the following steps: fatty acid transport, de novo fatty acid (FA) synthesis, FA synthesis, milk lipid synthesis, and finally droplet formation and secretion^51–53^. Fat production and milk FA composition depend on the stages of lactation and the level of milk production^52,54,55^. In general, most pathways enriched in the AP period of lactation contributed toward fat metabolism.

In addition, a number of key hub genes were identified in each module in the BP, P, and AP periods of lactation. In the P period, the *FUZ* gene had a higher intra-modular connectivity in the midnight-blue module, and this gene is involved in the hedgehog signaling pathway^56^. This pathway is known to be involved in the development process. Therefore, the regulation of the hedgehog pathway is necessary for the normal development of the offspring^56^.

Two different modules were significantly correlated with the P period, i.e., the green and tan modules. The main hub gene in the green module was the *YAP1* gene. Based on the co-expression network results of previous studies, the *YAP1* gene is involved in the module which is related to milk lactose^57^. In the tan module, the *CAMSAP3* gene was identified as the main hub gene. Previous genome-wide association studies (GWAS) on bovine populations highlighted the contribution of this gene in mastitis characterization^58^. Mastitis is recognized by a high cell count in the milk, which also happens to be one of the most important issues in the dairy industry in terms of economic losses^58^.

The main hub gene in the green–yellow module was the *P2RX3* gene. It has been proposed that *P2RX3* plays an important role in the immune system^59^. Prior studies have also confirmed that milk production results in a better functioning immune system^4^. The gene *EIF1AX* was identified as a main hub gene in the turquoise module, and we now know that this gene contributes to the synthesis of milk protein^60^.

## Materials and methods

### Data collection

The RNA-Seq datasets related to the lactation process were downloaded from the Gene Expression Omnibus (GEO) and European Nucleotide Archive (ENA) databases. Five RNA-Seq datasets for three different species, i.e., *Bos Taurus*, *Ovis aries,* and *Bubalus bubalis* (Table 4), were included in our study. Detailed information on the datasets is presented in Supplementary Table S1.

**Table 4:** Data set ID, species, and number of samples selected for meta-analysis.

Accession ID	Species	Reference	No. of samples			RNA source
			BP	P	AP	
SRP064718	Bos Taurus (Holestian-high milk production)	Yang, et al. (2014)	3	3	–	MFGs^1^
SRP125676	Bos Taurus	Bhat SA, et al. (2019)	3	2	3	MECs^2^
SRP065967	Ovis aries	Suárez-Vega, A., et al. (2016)	4	4	7	MSCs^3^
SRP144268	Bubalus bubalis (water buffalo)	Arora R., et al. (2019)	4	4	4	Milk
SRP153744	Bubalus bubalis (Murrah buffaloes)	Deng T., et al. (2019)	2	2	4	Biopsy

^1^MFGs = milk fat globules.

^2^Mammary epithelial cells = MECs.

^3^Milk Somatic Cells = MSCs.

**Figure 8:**
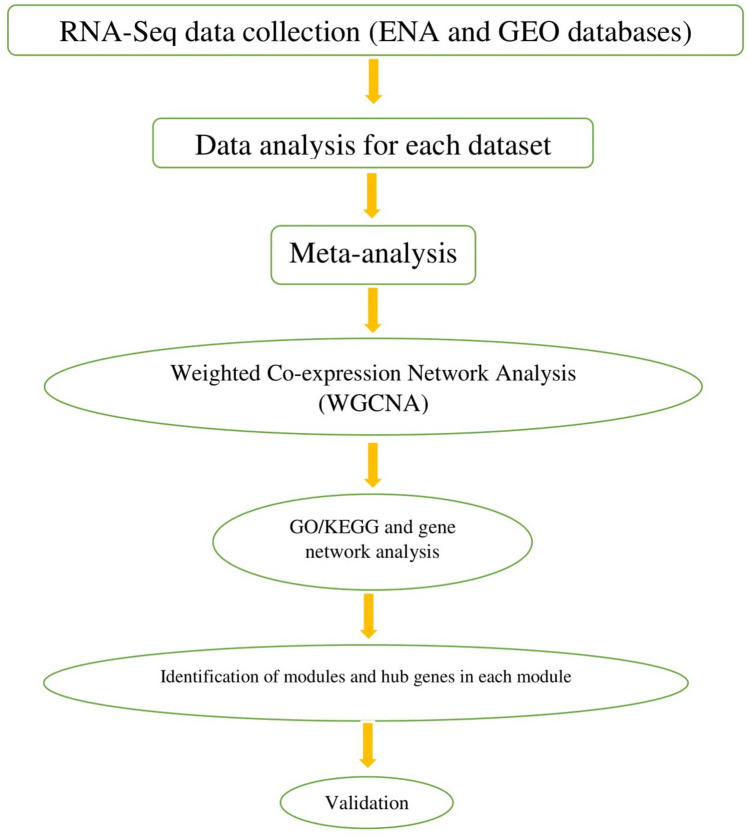
Flowchart of the performed meta-analysis and WGCNA analysis of the lactation process using the RNA-Seq datasets.

The first dataset (*SRP064718*) had 12 biological samples from six Chinese Holstein cows, which were divided into two groups, i.e., a high production group and a low production group. Samples in this dataset were collected at 10 days (n = 3) and 70 days (n = 3) after lactation, which were used as before peak and peak samples in the meta-analysis, respectively. The second dataset (*SRP125676*) covers mammary epithelial cells (MECs) at different stages of lactation (15, 90, and 250 days) from both Jersey and Kashmiri cattle. In this dataset, the samples from day 15 were included as before peak (Jersey = 3 and Kashmiri = 3 samples), from day 90 as peak (Jersey = 2 and Kashmiri = 3 samples), and from day 250 as the after peak group (Jersey = 3 and Kashmiri = 3 samples). The third dataset (*SRP065967*) covers milk somatic cells (MECs) from two dairy sheep breeds, i.e., Churra and Assaf. Milk samples were collected at the 10th, 50th, 120th, and 150th day of lactation. The samples obtained from each breed were treated as a separate dataset. Samples from the entire dataset were divided into three groups, i.e., before peak (day 10), peak (day 50), and after peak (days 120 and 150). The *SRP153744* dataset consisted of samples from Murrah buffaloes at four different stages of lactation, i.e., the 4th, 50th, 140th, and 280th day of lactation. The samples from day 4 were considered to be before peak lactation, day 50 samples were considered to be peak samples, and samples from the 140th and 280th days were considered to be in the after peak group. The fifth dataset (*SRP144268*) consisted of samples from buffalo milk. Four buffaloes were in each group (early, mid, and late lactation). The early, mid, and late stage samples were collected at 30–54, 117–136, and 250–273 days postpartum, respectively. All the steps of data collection and downstream analysis are presented in Fig. 8.

### RNA-Seq data processing

The quality of the raw data was assessed using FastQC (v 0.11.5) software^61^ and low quality reads were trimmed using the Trimmomatic (v 0.32) software^62^. The clean and trimmed read of sheep, cow, and buffalo were mapped onto the *Ovis aries* (*Oar_v4.0*), *Bos Taurus* (*Btau_5.0.1*), *Bubalus bubalis* (*UOA_WB_1*) reference genomes (available at www.ncbi.gov/genome), respectively, using Tophat (version2)^63^. Subsequently, The mapped reads from the BAM files were counted and assigned to each gene using the HTSeq-count^64^. To make an accurate comparison of gene expression between groups, the count values were first normalized. Then, differentially-expressed genes (DEGs) were screened using DESeq2^65^ (version 1.28.1) in R, using the Bioconductor package with default parameters. In this study, we used library size, size factor normalization factors, and group as covariate. The DESeq2 model internally corrects for library size; therefore, transformed or normalized values, such as counts scaled by library size, should not be used as input. The variance stabilizing transformations (VST)^66–68^ function was used to estimate the sample differences^65^. The VST function does not remove variation that can be associated with batch or other covariates. So, we used the “removeBatchEffect” function for remove batch variations^65^. Since it is not necessary to re-estimate the dispersion values, we used the blind = False option. The library size and other normalization factors have been normalized through this transformation. The samples belong each study were normalized together, means each dataset normalized separately.

The PCA plot for before and after normalization modes are presented in the Supplementary Table S2. The cutoff for differential expression was set at a fold change ≥|2| along with a corrected *p* value of ≤ 0.05^69^.

Since we used the gene lists for weighted co-express analysis using WGCNA packages which is designed for clustering genes based on their expression profiles. So, we filter genes which has a counts less than 10 in more than 90% of samples because these low expressed genes tend to reflect noise and correlations based on counts that are mostly zero aren’t really meaningful.

### Meta-analysis

In order to identify key genes in the lactation process, a meta-analysis was performed for the three stages of the lactation process. As data originally came from three different species, to check the effects of heterogeneous data sources on DEGs, ten attribute weighting algorithms were applied and the results showed the type of organism had no or little effect on the selected gene list^4^. First, BLAST pipeline was employed to identify the orthologous across three species^70^. Then, *p* values of differentially expressed genes in each dataset were calculated. Finally, *p* values were combined using the Fisher method, which was implemented in the metaRNA-Seq bioconductor package^71^. The combination of individual *p* values into one statistical test is defined as:

$${{\varvec{x}}}^{2}=-2\sum_{{\varvec{a}}=1}^{{\varvec{S}}}\mathbf{l}\mathbf{n}({{\varvec{p}}}_{{\varvec{g}}{\varvec{s}}})$$

where ${{\varvec{p}}}_{{\varvec{i}}}$ indicates the individual *p* value obtained from the gene, ${\varvec{g}}$ signifies the experiment, and ${\varvec{S}}$ is the total number of experiments. Based on the null hypothesis, the distribution of test statistic is ${{\varvec{x}}}^{2}$ with 2 degrees of freedom. Based on Table 1, we defined three stages of lactation (i.e., BF, P, and AP); therefore, three meta-analysis comparisons were performed as BP vs. P, BP vs. AP, and P vs. AP.

### Co-expression network construction

Meta-genes in each comparison (i.e., BP vs. P, BP vs. AP, and P vs. AP) were determined using the direct merging approach as described in a previous study^72^. Then, expression values of meta-genes were normalized and subjected to the WGCNA, using the Bioconductor R package (version 3.5.1)^20^, for weighted co-expression network construction. In summary, the similarity matrix between each pair of genes across all samples was calculated based on its Pearson’s correlation value. Then, the similarity matrix was transformed into an adjacency matrix. Subsequently, the topological overlap matrix (TOM) and the corresponding dissimilarity (1-TOM) value were computed. Finally, a dynamic tree cut (DTC) algorithm was employed to detect gene co-expression modules. The modules were constructed with a cut height of 0.975, and a minimum module size of 30 genes.

Protein–protein interaction (PPI) network of the identified modules was constructed based on the STRING database (https://string-db.org/)^73^ as prescribed by^74^. To visualize the constructed networks, the Cytoscape software (version 3.7.2)^75^ was used.

### Gene ontology analysis of significant modules

To interpret the biological significance of the DEGs, enrichment analysis was performed based on Gene Ontology and KEGG pathways^24^. ClueGO was used to illustrate overrepresented Gene Ontology (GO). ClueGO is a Cytoscape plug-in that visualizes the non-redundant biological terms for large numbers of genes, and integrates the GO terms to create a GO/pathway network^76^.

### Identification and validation of hub genes

Hub genes, defined as highly interconnected nodes in each module, are considered as functionally-important genes^77^. To identify the hub genes, the moduleEigengenes function was used for calculating the modules’ eigengenes, considered as the principal component of each module. Each network has several properties, including intramolecular connectivity (K_within_), total connectivity (K_total_), and module membership (ME), which can be used for the identification of genes with a high degree of connectivity within a module (i.e., hub genes)^78^. It is suggested that the hub genes may have a significant biological function within their module^78^.

In this study, the connectivity scores within the modules were calculated using between-centrality indices. The Cytoscape software (version 3.7.2)^75^ was used to visualize significant modules, and the hub genes in each corresponding module.

In order to validate and evaluate the hub genes’ efficiency for distinguishing different stages of lactation, the identified meta-genes with their corresponding expression values were subjected to feature (i.e., gene) selection based on ten weighting algorithms, i.e., PCA, Uncertainty, Relief, Chi Squared, Gini Index, Deviation, Rule, Gain Ratio, Information Gain, and Support-Vector Machines (SVM). Meta-genes with weighting values higher than 0.7 were selected for the construction of the Decision Tree (DT). The DTs were constructed using Information Gain, Information Gain ratio, Gini index, and Accuracy criteria along with the leave-one-out cross-validation (LOOCV) method. In this procedure, the initial dataset was split into a training set and a testing set. One sample from the initial dataset is consecutively discarded for the testing set, while the others remain for the training^25,79^.

The PRISMA checklist is included as Supplementary Table S3.

## Conclusions

In this study, we integrated a meta-analysis with the gene co-expression network analysis on RNA-Seq data to identify the key genes involved in the Before Peak (BP), Peak (P), and After Peak (AP) stages of the lactation process. The findings of this study highlighted the efficiency of the applied approaches for the identification of key genes and major pathways, involved in the lactation process. Enrichment analysis of the identified meta-genes highlighted the contribution of fat metabolism, cell differentiation, cell proliferation, milk protein production, and immune competency to the lactation process. Interestingly, all the above-mentioned functions affect milk quality and production. Furthermore, the findings of the current study support the notion that the WGCNA in combination with meta-analysis can provide an opportunity to obtain a better resolution analysis, which can better predict the most important functional genes that might provide a more robust bio-signature for phenotypic traits, thereby possibly providing more promising biomarker candidates for future studies.

## Supplementary Information

Supplementary file 1.

Supplementary file 2.

Supplementary file 3.

Supplementary file 4.

Supplementary file 5.

Supplementary file 6.

Supplementary file 7.

Supplementary file 8.

Supplementary file 9.

Supplementary table 1.

Supplementary table 2.

Supplementary table 3.
