# Author Correction: Weighted gene co-expression network analysis identifies modules and functionally enriched pathways in the lactation process

**DOI:** 10.1038/s41598-021-97893-1

**Published:** 2021-09-08

**Authors:** Mohammad Farhadian, Seyed Abbas Rafat, Bahman Panahi, Christopher Mayack

**Affiliations:** 1 Department of Animal Science, Faculty of Agriculture, University of Tabriz, Tabriz, Iran; 2 Department of Genomics, Branch for Northwest & West Region, Agricultural Biotechnology Research Institute of Iran (ABRII), Agricultural Research, Education and Extension Organization (AREEO), Tabriz, Iran; 3 Molecular Biology, Genetics, and Bioengineering, Faculty of Engineering and Natural Sciences, Sabancı University, Istanbul, 34956 Turkey

Correction to: *Scientific Reports* 10.1038/s41598-021-81888-z, published online 27 January 2021

The Acknowledgements section in the original version of this Article was incomplete.

“This research is supported by a research grant from the University of Tabriz (Number 3421).”

now reads:

“The authors would like to thank the Iran National Science Foundation (INSF, Grant No. 98023162) for financial support. This research is supported by a research grant from the University of Tabriz (Number 3421).”

The original Article has been corrected.

