# Supplementary material for: Weighted gene co-expression network analysis identifies modules and functionally enriched pathways in the lactation process
Source: Sci Rep. 2021 Jan 27;11:2367. doi: 10.1038/s41598-021-81888-z (PMC7840764; doi:10.1038/s41598-021-81888-z)
Supplement: Supplementary file 10 — Supplementary table 1. [file 41598_2021_81888_MOESM10_ESM.docx]

**Weighted gene co-expression network analysis identifies modules and functionally enriched pathways in the lactation process**

Mohammad Farhadian*^1^, Seyed Abbas Rafat^1^, Bahman Panahi^2^, Christopher Mayack^3^

1-Department of Animal Science, Faculty of Agriculture, University of Tabriz, Tabriz, Iran

2 -Department of Genomics, Branch for Northwest & West region, Agricultural Biotechnology Research Institute of Iran (ABRII), Agricultural Research, Education and Extension Organization (AREEO), Tabriz, Iran

3 - Molecular Biology, Genetics, and Bioengineering, Faculty of Engineering and Natural Sciences, Sabancı University, Istanbul, 34956, Turkey

***Corresponding author:**

Mohammad Farhadian, Department of Animal Science, Faculty of Agriculture, University of Tabriz, Tabriz, Iran.

Tel: +98 9149765639

Email: [Mohammad.farhadian@tabrizu.ac.ir](mailto:Mohammad.farhadian@tabrizu.ac.ir)

**Supplementary Table S1.** Detailed information of the datasets

| Accession ID | Species | Stage | Run Accession | Read Count | mapping rate |
| --- | --- | --- | --- | --- | --- |
| SRP064718 | Bos Taurus (Holestian- high milk production group) | BP | SRR2632970 | 37149199 | 94.2% |
|  |  |  | SRR2641154 | 30812254 | 96.4% |
|  |  |  | SRR2641155 | 32894031 | 97.0% |
|  |  | P | SRR2633596 | 42642767 | 96.3% |
|  |  |  | SRR2641157 | 39843947 | 91.5% |
|  |  |  | SRR2641159 | 31166382 | 96.6% |
| SRP064718 | Bos Taurus (Holestian- low milk production group) | BP | SRR2634907 | 46916030 | 96.2% |
|  |  |  | SRR2641161 | 33403183 | 95.8% |
|  |  |  | SRR2641163 | 33567788 | 95.7% |
|  |  | P | SRR2635009 | 30140101 | 97.5% |
|  |  |  | SRR2641165 | 30674810 | 96.3% |
|  |  |  | SRR2641166 | 30102981 | 96.6% |
| SRP125676 | Bos Taurus (Jersy) | BP | SRR6324365 | 48124770 | 85.7% |
|  |  |  | SRR6324366 | 45607023 | 82.1% |
|  |  |  | SRR6324367 | 45302553 | 82.7% |
|  |  | P | SRR6324368 | 46258686 | 89.1% |
|  |  |  | SRR6324369 | 46617564 | 88.2% |
|  |  | AP | SRR6324370 | 58200765 | 91.0% |
|  |  |  | SRR6324371 | 68414217 | 91.5% |
|  |  |  | SRR6324372 | 38824961 | 90.0% |
| SRP125676 | Bos Taurus (Kashmiri ) | BP | SRR6324373 | 41231387 | 79.7% |
|  |  |  | SRR6324374 | 40905980 | 76.7% |
|  |  |  | SRR6324375 | 52285576 | 79.7% |
|  |  | P | SRR6324376 | 36943668 | 90.0% |
|  |  |  | SRR6324377 | 34212918 | 85.4% |
|  |  |  | SRR6324378 | 38487367 | 90.4% |
|  |  | AP | SRR6324379 | 46049214 | 93.1% |
|  |  |  | SRR6324379 | 43860412 | 91.1% |
|  |  |  | SRR6324381 | 60444037 | 90.9% |

| Accession ID | Species | Stage | Run Accession | Read Count | mapping rate |
| --- | --- | --- | --- | --- | --- |
| SRP065967 | [Ovis aries](https://www.ebi.ac.uk/ena/data/view/Taxon:Ovis%20aries) (Assaf) | BP | [SRR2932535](https://www.ebi.ac.uk/ena/data/view/SRR2932535) | 35043772 | 84.5% |
|  |  |  | [SRR2932536](https://www.ebi.ac.uk/ena/data/view/SRR2932536) | 33159147 | 86.7% |
|  |  |  | [SRR2932537](https://www.ebi.ac.uk/ena/data/view/SRR2932537) | 31081902 | 86.1% |
|  |  |  | [SRR2932538](https://www.ebi.ac.uk/ena/data/view/SRR2932538) | 41322109 | 87.4% |
|  |  | P | SRR2932543 | 40313876 | 88.5% |
|  |  |  | SRR2932544 | 42580059 | 88.8% |
|  |  |  | SRR2932545 | 29793742 | 84.9% |
|  |  |  | SRR2932546 | 34921005 | 89.4% |
|  |  | AP | SRR2932551 | 41394140 | 88.1% |
|  |  |  | SRR2932552 | 38933690 | 87.8% |
|  |  |  | SRR2932553 | 36187490 | 89.6% |
|  |  |  | SRR2932557 | 37213653 | 88.6% |
|  |  |  | SRR2932558 | 29991596 | 87.5% |
|  |  |  | SRR2932559 | 40861735 | 89.3% |
|  |  |  | SRR2932560 | 35432575 | 89.2% |
| SRP065967 | [Ovis aries](https://www.ebi.ac.uk/ena/data/view/Taxon:Ovis%20aries) (Churra) | BP | [SRR2932539](https://www.ebi.ac.uk/ena/data/view/SRR2932538) | 30130200 | 87.8% |
|  |  |  | [SRR2932540](https://www.ebi.ac.uk/ena/data/view/SRR2932538) | 28025028 | 84.9% |
|  |  |  | [SRR2932541](https://www.ebi.ac.uk/ena/data/view/SRR2932538) | 36170492 | 88.3% |
|  |  |  | [SRR2932542](https://www.ebi.ac.uk/ena/data/view/SRR2932538) | 23297744 | 86.3% |
|  |  | P | SRR2932547 | 29180331 | 86.9% |
|  |  |  | SRR2932548 | 45690280 | 90.1% |
|  |  |  | SRR2932549 | 45967224 | 88.0% |
|  |  |  | SRR2932550 | 42476719 | 90.3% |
|  |  | AP | SRR2932554 | 35170966 | 89.6% |
|  |  |  | SRR2932555 | 39951447 | 87.3% |
|  |  |  | SRR2932556 | 33519563 | 89.5% |
|  |  |  | SRR2932561 | 31562685 | 89.6% |
|  |  |  | SRR2932562 | 42943369 | 87.3% |
|  |  |  | SRR2932563 | 35600309 | 90.9% |
|  |  |  | SRR2932564 | 43217874 | 89.9% |

| Accession ID | Species | Stage | Run Accession | Read Count | mapping rate |
| --- | --- | --- | --- | --- | --- |
| SRP153744 | Bubalus bubalis (Murrah) | BP | [SRR7523532](https://www.ebi.ac.uk/ena/data/view/SRR7523531) | 23453726 | 88.5% |
|  |  |  | [SRR7523533](https://www.ebi.ac.uk/ena/data/view/SRR7523531) | 23051139 | 88.8% |
|  |  | P | [SRR7523531](https://www.ebi.ac.uk/ena/data/view/SRR7523531) | 23099977 | 84.9% |
|  |  |  | SRR7523534 | 23469189 | 87.8% |
|  |  | AP | SRR7523535 | 23436067 | 88.0% |
|  |  |  | SRR7523536 | 23592761 | 90.3% |
|  |  |  | SRR7523537 | 22970160 | 89.6% |
|  |  |  | SRR7523538 | 22804966 | 90.4% |
| SRP144268 | Bubalus bubalis ( water buffalo) | BP | [SRR7091387](https://www.ebi.ac.uk/ena/data/view/SRR7091387) | 36922575 | 87.4% |
|  |  |  | [SRR7091388](https://www.ebi.ac.uk/ena/data/view/SRR7091387) | 49030291 | 88.5% |
|  |  |  | [SRR7091389](https://www.ebi.ac.uk/ena/data/view/SRR7091387) | 23886844 | 88.8% |
|  |  |  | SRR7091390 | 31626239 | 84.9% |
|  |  | P | SRR7091391 | 43354873 | 89.4% |
|  |  |  | SRR7091392 | 44463066 | 88.1% |
|  |  |  | SRR7091393 | 52864049 | 89.7% |
|  |  |  | SRR7091394 | 40489352 | 91.03% |
|  |  | AP | SRR7091395 | 24054842 | 88.2% |
|  |  |  | SRR7091396 | 54377304 | 89.5% |
|  |  |  | SRR7091397 | 34821589 | 90.1% |
|  |  |  | SRR7091398 | 57139131 | 88.7% |

BP: before peak, P: peak, AP: after peak
