# Supplementary material for: Weighted gene co-expression network analysis identifies modules and functionally enriched pathways in the lactation process
Source: Sci Rep. 2021 Jan 27;11:2367. doi: 10.1038/s41598-021-81888-z (PMC7840764; doi:10.1038/s41598-021-81888-z)
Supplement: Supplementary file 11 — Supplementary table 2. [file 41598_2021_81888_MOESM11_ESM.docx]

**Weighted gene co-expression network analysis identifies modules and functionally enriched pathways in the lactation process**

Mohammad Farhadian*^1^, Seyed Abbas Rafat^1^, Bahman Panahi^2^, Christopher Mayack^3^

1-Department of Animal Science, Faculty of Agriculture, University of Tabriz, Tabriz, Iran

2 -Department of Genomics, Branch for Northwest & West region, Agricultural Biotechnology Research Institute of Iran (ABRII), Agricultural Research, Education and Extension Organization (AREEO), Tabriz, Iran

3 - Molecular Biology, Genetics, and Bioengineering, Faculty of Engineering and Natural Sciences, Sabancı University, Istanbul, 34956, Turkey

***Corresponding author:**

Mohammad Farhadian, Department of Animal Science, Faculty of Agriculture, University of Tabriz, Tabriz, Iran.

Tel: +98 9149765639

Email: [Mohammad.farhadian@tabrizu.ac.ir](mailto:Mohammad.farhadian@tabrizu.ac.ir)

**Supplementary Table S2.** The PCA plot for before and after normalization

| 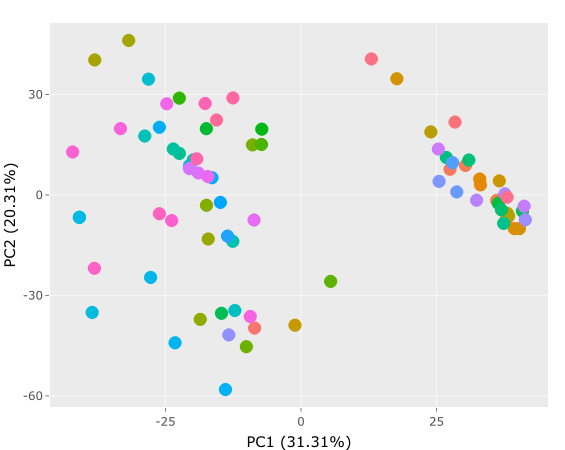 | 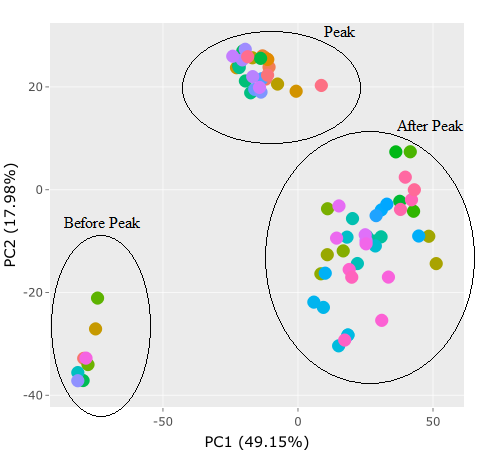 |
| --- | --- |
| A | B |

A: Before normalization

B: After normalization
