# Supplementary material for: Weighted gene co-expression network analysis identifies modules and functionally enriched pathways in the lactation process
Source: Sci Rep. 2021 Jan 27;11:2367. doi: 10.1038/s41598-021-81888-z (PMC7840764; doi:10.1038/s41598-021-81888-z)
Supplement: Supplementary file 12 — Supplementary table 3. [file 41598_2021_81888_MOESM12_ESM.docx]

**Weighted gene co-expression network analysis identifies modules and functionally enriched pathways in the lactation process**

Mohammad Farhadian*^1^, Seyed Abbas Rafat^1^, Bahman Panahi^2^, Christopher Mayack^3^

1-Department of Animal Science, Faculty of Agriculture, University of Tabriz, Tabriz, Iran

2 -Department of Genomics, Branch for Northwest & West region, Agricultural Biotechnology Research Institute of Iran (ABRII), Agricultural Research, Education and Extension Organization (AREEO), Tabriz, Iran

3 - Molecular Biology, Genetics, and Bioengineering, Faculty of Engineering and Natural Sciences, Sabancı University, Istanbul, 34956, Turkey

***Corresponding author:**

Mohammad Farhadian, Department of Animal Science, Faculty of Agriculture, University of Tabriz, Tabriz, Iran.

Tel: +98 9149765639

Email: [Mohammad.farhadian@tabrizu.ac.ir](mailto:Mohammad.farhadian@tabrizu.ac.ir)


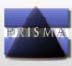
**PRISMA 2009 Checklist**

| **Section/topic** | **#** | **Checklist item** | **Reported on page #** |
| --- | --- | --- | --- |
| **TITLE** | | |  |
| Title | 1 | Meta-analysis of Transcriptional data in milk production process. | 1 |
| **ABSTRACT** | | |  |
| Structured summary | 2 | **Note: This is NOT a meta-analysis of clinical or preclinical trials**.  Some items in this list will not be applicable. This is a meta-analysis of data in RNA-Seq transcriptional profiling studies as available in public repositories. | 3 |
| **INTRODUCTION** | | |  |
| Rationale | 3 | Meta-analysis with increase the statistical power and the generalizability of single-study analysis, can bypass the challenges associated with individual variations, and robust to the mildest data perturbations. | 3.4 |
| Objectives | 4 | PICOS Participants: Individual cows, buffalo and sheep milk production data. Interventions: None. Comparisons: Before peak vs. Peak; Before peak vs. After peak; Peak vs. After peak. Outcomes: Not applicable. Study design: mRNA analysis using RNA-Seq data. | 3.4 |
| **METHODS** | | |  |
| Protocol and registration | 5 | No review protocol | N/A |
| Eligibility criteria | 6 | Not applicable | N/A |
| Information sources | 7 | EBI datasets | 3 |
| Search | 8 | Keywords: Bos Taurus, [Ovis aries](https://www.ebi.ac.uk/ena/data/view/Taxon:Ovis%20aries), Bubalus bubalis, Milk, RNA-seq | 3 |
| Study selection | 9 | The studies which include samples in Before Peak, Peak and After peak stage were selected for meta-analysis | 3 |
| Data collection process | 10 | Downloading RAW fastq file from EBI database. | 3 |
| Data items | 11 | Not applicable | N/A |
| Risk of bias in individual studies | 12 | Not applicable | N/A |
| Summary measures | 13 | Not applicable | N/A |
| Synthesis of results | 14 | Not applicable | N/A |
